# Énorme adénopathie cervicale révélant un lymphome non hodgkinien du nasopharynx

**DOI:** 10.11604/pamj.2020.37.376.17562

**Published:** 2020-12-23

**Authors:** Ahmed Rouihi, Bouchaib Hemmaoui

**Affiliations:** 1 Service d’Otorhinolaryngologie et de Chirurgie Cervico-Faciale de l’Hôpital Militaire d’Instruction Mohamed V de Rabat, Faculté de Médecine et Pharmacie Rabat, Université Mohamed V Rabat, Rabat, Maroc

**Keywords:** Lymphome non hodgkinien, nasopharynx, adénopathie, non-Hodgkin lymphoma, nasopharynx, lymphadenopathy

## Abstract

Non-Hodgkin's lymphoma is a rare histological type of nasopharyngeal cancer. Most of these cancers are undifferentiated carcinomas or undifferentiated nasopharyngeal carcinomas (UNC). We report a case of nasopharyngeal non-Hodgkin's lymphoma. Immunohistochemical examination showed B-cell non-Hodgkin's lymphoma. The study involved a 47-year old patient with no particular past medical history presenting with right laterocervical swelling associated with right nasal obstruction and rhinorrhea that had persisted for 6 months. Clinical examination showed right voluminous painless adherent laterocervicale lymphadenopathy without signs of inflammation. Endoscopic examination of the nasopharynx showed enormous process extending to the posterior nares. Anatomo-pathologic study of the biopsy with immunohistochemical study showed B-cell non-Hodgkin's lymphoma. Patient's outcome was favorable under chemotherapy with no relapse during 6-month follow-up period. Primary non-Hodgkin's lymphoma of the nasopharynx is a rare localization of lymphomas. It occurs in less than 10% of patients with lymphoma of the head and of the neck. It often poses a problem in positive clinical and histological diagnosis. Symptoms are generally little specific. Peripheral lymphadenopathies occur in 50% of cases. Diagnosis is based on biopsy with immunohistochemical examination. Therapeutic progress have been achieved through the more frequent use of chemotherapy associated with radiotherapy.

## Image in médicine

Le lymphome malin non hodgkinien est une entité histologique rare parmi les cancers du nasopharynx, la plupart des tumeurs étant des carcinomes indifférenciés ou Undifferentiated Carcinoma of Nasopharyngeal Type (UCNT). Il pose souvent un problème de diagnostic positif clinique et histologique. Nous rapportons le cas d'un lymphome non hodgkinien du nasopharynx, l'analyse immunohistochimique était en faveur d'un lymphome malin non hodgkinien de phénotype B. Il s´agit d´un patient âgé de 47ans, sans antécédents pathologiques particuliers, qui présente depuis 6 mois une tuméfaction laterocervicale droite avec obstruction nasale droite et rhinorrhée. L´examen clinique a trouvé une grosse adénopathie laterocervicale droite adhérente, indolore sans signes d´inflammation en regard. L´examen endoscopique du nasopharynx a retrouvé un énorme processus arrivant jusqu´aux choanes. L´étude anatomopathologique de la biopsie avec étude immunohistochimique était en faveur d´un lymphome malin non hodgkinien de phénotype B. L´évolution était favorable sous chimiothérapie avec un recul de 6 mois sans signes de rechute. Le lymphome non hodgkinien primitif du nasopharynx est une localisation rare des lymphomes. Il survient chez moins de 10% des patients atteints de lymphome de la tête et du cou. Il pose souvent un problème de diagnostic positif clinique et histologique. La symptomatologie est généralement peu spécifique, les adénopathies périphériques sont présente dans 50% des cas. Le diagnostic repose sur la biopsie avec étude immunohistochimique. Des progrès thérapeutiques ont été obtenus par l´utilisation plus fréquente de la chimiothérapie associée à la radiothérapie.

**Figure 1:**
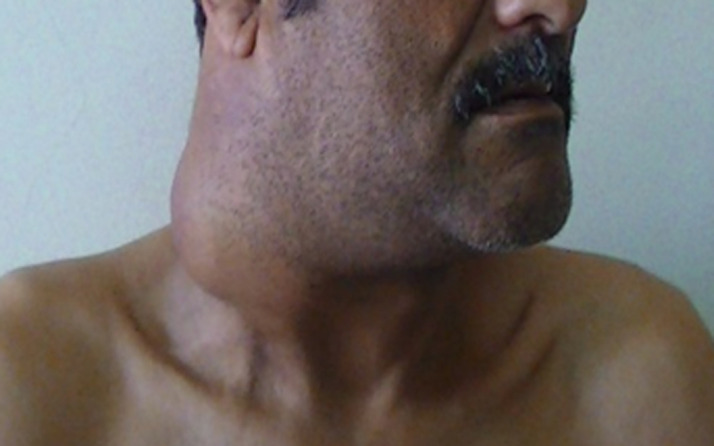
image objectivant une énorme tuméfaction latéro-cervicale droite

